# Patient safety culture lives in departments and wards: Multilevel partitioning of variance in patient safety culture

**DOI:** 10.1186/1472-6963-10-85

**Published:** 2010-03-31

**Authors:** Ellen Deilkås, Dag Hofoss

**Affiliations:** 1 Health Services Research Unit, Akershus University Hospital, Norway

## Abstract

**Background:**

Aim of study was to document 1) that patient safety culture scores vary considerably by hospital department and ward, and 2) that much of the variation is across the lowest level organizational units: the wards. Setting of study: 500-bed Norwegian university hospital, September-December 2006.

**Methods:**

Data collected from 1400 staff by (the Norwegian version of) the generic version of the Safety Attitudes Questionnaire (SAQ Short Form 2006). Multilevel analysis by MLwiN version 1.10.

**Results:**

Considerable parts of the score variations were at the ward and department levels. More organization level variation was seen at the ward level than at the department level.

**Conclusions:**

Patient safety culture improvement efforts should not be limited to all-hospital interventions or interventions aimed at entire departments, but include involvement at the ward level, selectively aimed at low-scoring wards. Patient safety culture should be studied as closely to the patient as possible. There may be such a thing as "hospital safety culture" and the variance across hospital departments indicates the existence of department safety cultures. However, neglecting the study of patient safety culture at the ward level will mask important local variations. Safety culture research and improvement should not stop at the lowest formal level of the hospital (wards, out-patient clinics, ERs), but proceed to collect and analyze data on the micro-units within them.

## Background

Although the risk of harming patients is evident to most caregivers, eliminating or reducing risk has not always been the first priority of health care management. Management often takes the safety of patients for granted, and considers patient safety as the responsibility of caregivers honouring the guideline "primum non nocere".

Compared to other Scandinavian countries, Norway has only recently made patient safety a national health policy issue, following a series of non-governmental calls for action, most notably by professor emeritus Peter F. Hjort's 2004 policy suggestion [1] and 2007 textbook [2]. In 2007 the Directorate of Health established a national unit for patient safety and in 2009 the Ministry of Health launched a national patient safety campaign.

Efforts to improve patient safety may follow several lines of action, including mortality-and-morbidity conferences, sentinel event scrutiny, restructuring of care delivery systems and safety culture surveys [3]. Each strategy has its merits, and surveying safety culture is a useful option, not least because a common experience in patient safety improvement work is that interventions directed against specific causes of adverse events often result in only temporary improvement. One possible interpretation is that adverse events have multiple causes, and the quintessential explanation is the priority of safety reflected in the general patient safety culture of the unit in which the adverse events occur.

The aim of the study was to test the hypothesis that patient safety culture is a local phenomenon, implying that patient safety culture scores vary considerably by hospital department and ward, and that much of the variation is across the lowest level organizational units: the wards.

## Methods

### Study design

This study analyzes patient safety attitudes data on clinical staff at Akershus University Hospital. Responses of staff to a patient safety attitudes questionnaire were collected from October to December 2006. If our hypothesis was correct, we would expect to find considerable clustering of the safety attitude scores at the department and ward level, i.e. non-trivial-to-high intraclass correlation coefficients.

The study plans were presented to the Regional Ethical Committee for Medical Research in Eastern Norway for approval. The Committee decided the project did not require their approval as it did not involve collecting data on patients. The collection of data on clinical staff was approved by the proper government authority, the Norwegian Data Inspectorate.

### Setting and participants

Akershus University Hospital is located just outside Oslo, the capital of Norway. In 2006 the hospital had 500 somatic and 200 psychiatric beds, 4200 employees, and an annual budget of 2.500.000.000 NOK (approximately 450 million USD). It is a general hospital with a wide variety of specialities, but it does not include an eye clinic and a geriatric department. In 2006 it served a population of 280 000 inhabitants of Northeast Oslo and the Northeastern part of the Oslo-surrounding county of Akershus. It treated 53.000 inpatients and had 150.000 outpatient consultations. Eighty-five percent of the inpatients were unscheduled emergency cases.

The questionnaire was distributed to all clinical staff (physicians, registered nurses, auxiliary nurses, radiographers, laboratory technicians, midwives, and clerical workers) at 45 somatic caregiving units - 27 wards, 14 outpatient service units, and four laboratories - of 10 clinical departments: emergency admissions, anesthesiology, surgery, operations, orthopedics, gynecology and obstetrics, pediatrics, internal medicine, neurology and ear-nose-throat.

### The survey

The survey instrument used was (the Norwegian translation of) the Safety Attitudes Questionnaire (the SAQ) Short Form 2006. The original (American) version of the questionnaire is described by Sexton, Helmreich, Neilands et al. [4]. The Norwegian version of the SAQ has 41 questions, of which 36 reflect seven patient safety culture dimensions: Team Climate, Safety Climate, Job Satisfaction, Stress Recognition, Perception of Unit Management, Perception of Hospital Management and Work Conditions. The items which reflect each dimension are listed in Deilkås & Hofoss [5], which also describes the development of the Norwegian version of the SAQ and the assessment of its psychometric properties.

### Data collection

The survey was carried out at the hospital's somatic clinical areas during October-December 2006.

The questionnaires, which took approximately 15-20 minutes to complete, were distributed and completed at regular staff meetings. Forms were to be completed anonymously and did not have an ID number which could be used to trace the responder. Employees who did not attend the staff meeting were sent their SAQs through the hospital's internal mail system. As the questionnaires were anonymous, we had no way of reminding non-responders, except for asking the ward and department heads to urge their staff to participate.

### Statistical analysis

To calculate the factor scores, we reversed the scores on the negatively worded items (2 and 11). For each factor, the mean of the item scores was calculated. One was subtracted from each mean, and the result was multiplied by 25.

To partition the variation of the dimension scores by organization level, the seven patient safety culture scores were analyzed by MLwiN, a multilevel analysis program developed by the University of London's Institute of Education [6]. The program is now being distributed and expanded by the University of Bristol's Centre for Multilevel Modelling [7]. Multilevel analysis makes it possible to partition the total variance in each dimension score into variance across individual respondents (individual level variance), variance across wards (ward level variance) and variance across departments (department level variance). Analyzing the model which contains only the intercept (the data set's average patient safety attitudes score) and no explanatory variables - what is known as "the empty model" [8] - one can calculate the percentage of the total variance in patient safety attitudes scores that reside at the organizational level, that is, the percentage of the variance which is not score differences across individual responders, but across the organizational units.

The ratio of the variance at the organizational level to the total variance in the data is the intraclass correlation coefficient (ICC). Multiplied by 100, the ICC can be interpreted as the percentage of the total variance in the data set which belongs at the organizational level, that is, the percentage of the variance that is not differences across individual responders, but across the organizational units. By defining the multilevel model as having three levels - employee, ward and department - the MlwiN output showed how much the individual scores varied around the grand mean of each dimension score, as well as the variance in ward averages and department averages. The null hypothesis was that there was no clustering by organization level in the response data, implying that there were no differences among wards or among departments, i.e. all of the variance was across individual responders, and no ward or department stood out as a more promising candidate for patient safety improvement than any other ward or department. Our alternative hypothesis was that our data would show significant differences across wards and across departments, implying that patient safety improvement work should not address all departments and wards with the same reforms, but focus on the specific problems in units with lower scores. The results are shown in Table 1.

**Table 1:** Organization level variance by patient safety attitudes dimension

Dimension (all dimensions scaled 0-100)	Total variance	Variance at individual level (% of total variance)	Variance at ward level (% of total variance)	Variance at department level (% of total variance)	ICC (ratio of organizational level variance to total variance)
Teamwork Climate (valid n: 1090)	285,365	231,298 (81,1%)	39,245 (13,8%)	14,822 (5,2%)	0,19
Safety Climate (valid n: 984)	240,638	206,303 (85,7%)	21,733 (9,0%)	12,602 (5,2%)	0,14
Job Satisfaction (valid n: 1036)	365,350	309,274 (84,7%)	28,081 (7,7%)	27,995 (7,7%)	0,15
Stress Recognition (valid n: 1024)	491,506	483,168 (98,3%)	1,140 (0,2%)	7,198 (1,5%)	0,02
Work Conditions (valid n: 843)	411,830	352,886 (85,7%)	20,704 (5,0%)	38,240 (9,3%)	0,14
Perception of Unit Management (valid n: 949)	519,785	412,491 (79,4%)	68,706 (13,2%)	38,588 (7,4%)	0,21
Perception of Hospital Management (valid n: 904)	373,291	347,452 (93,1%)	12,430 (3,3%)	13,409 (3,6%)	0,07

The statistical significance of the variance at organization levels was judged by the change in the goodness-of-fit of the model to the data, as measured by the change in the model's log likelihood ratio produced by eliminating that level from the model. Judging significance by the ratio of the parameter estimate to its standard error works quite well for fixed parameters, that is, parameters estimated under the assumption of having the same value in all subunits of the data set. For random parameters, however, the distribution of this ratio may depart considerable from normality, and a better test for random parameters is to use the likelihood ratio statistic [9]. In our case, the "large sample" distribution of the -2LL-value under the null hypothesis (H_0 _= the two-level model is adequate) is a χ^2^-distribution with k_2_-k_1 _degrees of freedom - that is: d.f. = 3-2 = 1. The critical value for p <,05 for the change in -2LL is 3,84; for p <,01, it is 5,99. As suggested by Pinheiro & Bates (10), this test can be conservative, producing from the χ^2^_k2-k1 _distribution a p-value which is greater than it should be. What we did, then, was to respecify our models, removing from them the idea that there was variation across department to see how much - if at all - the respecification damaged the three-level models' goodness-of-fit. If we were correct in assuming significant score variation at all three levels, the two-level model would prove a worse fit to the data than the three-level model. The results are shown in Table 2.

**Table 2:** Organization level variance by patient safety attitudes dimension

Dimension	-2LL of three-level Model	-2LL of two-level model (individuals & wards)	Change in -2LL when department level was removed from model
Teamwork Climate	9103,317	9103,477	0,163 (n.s.: p > ,05)
Safety Climate	8095,995	8101,995	5,532 (p < ,05)
Job Satisfaction	8940,755	8946,288	5,533 (p < ,05)
Stress Recognition	9245,302	9248,427	3,125 (n.s.: p > ,05)
Work Conditions	7283,521	7289,960	6,439 (p < ,01)
Perception of Unit Management	8482,990	8484,253	1,263 (n.s.: p > ,05)
Perception of Hospital Management	7887,215	7890,124	2,809 (n.s.: p > ,05)

## Results

All clinical staff, a total of 1911, were asked to complete the SAQ, and 1306 (68%) did. The response rate was higher among nurses, auxiliary nurses, midwives, laboratory technicians, radiographers, physiotherapists and other staff with less education (as compared to the physicians). Response rates were higher (98 percent) among those who received their SAQs at staff meetings. Further details on response rates are published in Deilkås & Hofoss [5].

As shown in Table 1 five of the seven patient safety dimension scores showed considerable variance at the organizational level. Except for Stress Recognition (ICC =,02) and Perception of Hospital Management (ICC =,07) all dimensions had ICCs of 14 percent or higher. The highest ICC value was for Perception of Unit Management (21 percent) and Teamwork Climate (19 percent). For the dimension Work Conditions, clustering was more pronounced at the department level than at the ward level. For the dimensions of Teamwork Climate, Safety Climate and Perception of Unit Management, clustering was more pronounced at the ward level.

As shown in Table 2, for four of the seven dimensions - Teamwork Climate, Stress Recognition, Perception of Unit Management and Perception of Hospital Management - the elimination of the department level from the model did not reduce the model's goodness-of-fit significantly, as measured by the change in the -2LL. For the remaining three dimensions - Safety Climate, Job Satisfaction, and Work Conditions - the exclusion of the department level from the model did worsen the model's goodness-of-fit.

## Discussion

Given that hospital top management wish to improve patient safety culture, where should they intervene? Obviously, patient safety culture scores depend on the personal interest, attention and engagement of each staff member. The major part of the variance in patient safety attitudes was across individual employees, so efforts to promote a patient safety culture must continue targeting individual staff members. But we also found marked clustering of patient safety culture scores at the organizational levels, and much of the organization level variance was across wards. In some patient safety culture dimensions department averages differ, but in other dimensions, wards vary more strongly than departments. Therefore, interventions to improve patient safety should aim not only at individual employees, but also at organizational units, in particular those at the sharpest end of the health services: the wards.

Having data on one hospital only, we have not been able to check empirically the amount of clustering of safety attitudes at the hospital level, but, as indicated by Sexton, Helmreich, Neilands et al. [4], there probably are hospital-specific patient safety cultures. However, as shown by Pronovost & Sexton [11] and by Singer [12], variability in SAQ measurements may be greater across working groups than across hospitals. This analysis adds to the suggestion that strategies for improving safety climate and patient safety should be tailored for work areas and disciplines by estimating the relative size of the variances at ward and department levels for each of the seven safety attitudes dimensions.

As we have documented significant clustering of three patient safety attitude dimensions at the department level (safety climate, job satisfaction, and work conditions), it should be noted that there may be differences in patient safety culture across departments. However, for four of the seven dimensions, there was no evidence of variation across departments, only across wards.

Patient safety culture improvement efforts should, therefore, include interventions at the ward level, and not just department or all-hospital interventions. Zohar et al. [13] has reported how information on safety climate has been used to guide prevention efforts toward selected units. Selection must, however, be done with discretion in order to avoid stigmatizing working units as "low-score." And one must not focus solely on the low scorers: high-scoring units may also be interesting; lessons may be learnt from their successes.

Possibly, even probably, one should in the future also aim at studying even lower-level units, the "micro-systems" that do not appear in organizational blueprints, but in which so much of the actual clinical work is carried out [14-16]. The importance of studying such lower-level units is obvious enough in medical departments. One may easily see patient safety as a function of the safety culture of sub-groups of nurses or small nurse-doctor groups within a ward. The point is particularly obvious in surgical departments, where the wards are the bed units where patients are prepared for surgery and nursed after having undergone surgery, but the work that gives the department its name - and is vital to surgical patients' safety - takes place in the theatres of the department's operating section. Studying surgical department patient safety at the ward level, although bedside, one might easily miss important information. A data collection problem is that micro-systems like operating teams are temporary groups, which do not have permanently designated staff. This may differ among organizations: at our hospital operation teams are temporary, but in other organizations they may be permanent. The inclusion of the micro-unit level into multi-level analyses of patient safety attitudes and other aspects of patient safety is an important task for future patient safety research.

## Conclusions

1) Patient safety culture should be studied in care-giving units as close to the patient as possible. There may be such a thing as "hospital safety culture," and there are differences across hospital departments. However, neglecting the study of patient safety culture at the ward level will mask important local variations.

2) Patient safety culture improvement efforts should include interventions at ward level, not just department or all-hospital interventions.

3) Future research should not stop at the level of hospital wards, out-patient clinics, and ERs, but collect and analyze data on the micro-systems within them: nurse teams, doctor-nurse teams, operating teams etc.

## Competing interests

The authors declare that they have no competing interests.

## Authors' contributions

The two authors jointly designed the study, collected, analyzed and interpreted the data and wrote the manuscript.

## Pre-publication history

The pre-publication history for this paper can be accessed here:

http://www.biomedcentral.com/1472-6963/10/85/prepub
